# Shoot transcriptome revealed widespread differential expression and potential molecular mechanisms of chickpea (*Cicer arietinum* L.) against *Fusarium* wilt

**DOI:** 10.3389/fmicb.2023.1265265

**Published:** 2024-02-01

**Authors:** Karma L. Bhutia, Mahtab Ahmad, Anima Kisku, R. A. Sudhan, Nangsol D. Bhutia, V. K. Sharma, Bishun Deo Prasad, Mahendar Thudi, Oliver Obročník, Viliam Bárek, Marian Brestic, Milan Skalicky, Ahmed Gaber, Akbar Hossain

**Affiliations:** ^1^Department of Agricultural Biotechnology and Molecular Biology, CBS&H, Dr. Rajendra Prasad Central Agricultural University, Pusa, Bihar, India; ^2^College of Horticulture and Forestry, Central Agricultural University (Imphal), Pasighat, Arunachal Pradesh, India; ^3^Department of Water Resources and Environmental Engineering, Faculty of Horticulture and Landscape Engineering, Slovak University of Agriculture, Nitra, Slovakia; ^4^Institute of Plant and Environmental Sciences, Slovak University of Agriculture, Nitra, Slovakia; ^5^Department of Botany and Plant Physiology, Faculty of Agrobiology, Food, and Natural Resources, Czech University of Life Sciences Prague, Prague, Czechia; ^6^Department of Biology, College of Science, Taif University, Taif, Saudi Arabia; ^7^Division of Soil Science, Bangladesh Wheat and Maize Research Institute, Dinajpur, Bangladesh

**Keywords:** chickpea, *Fusarium* wilt, RNA sequencing, transcriptome, environmental adaptation, energy metabolism

## Abstract

**Introduction:**

The yield of chickpea is severely hampered by infection wilt caused by several races of *Fusarium oxysporum* f. sp. *ciceris (Foc).*

**Methods:**

To understand the underlying molecular mechanisms of resistance against *Foc4 Fusarium* wilt, RNA sequencing-based shoot transcriptome data of two contrasting chickpea genotypes, namely KWR 108 (resistant) and GL 13001 (susceptible), were generated and analyzed.

**Results and Discussion:**

The shoot transcriptome data showed 1,103 and 1,221 significant DEGs in chickpea genotypes KWR 108 and GL 13001, respectively. Among these, 495 and 608 genes were significantly down and up-regulated in genotypes KWR 108, and 427 and 794 genes were significantly down and up-regulated in genotype GL 13001. The gene ontology (GO) analysis of significant DEGs was performed and the GO of the top 50 DEGs in two contrasting chickpea genotypes showed the highest cellular components as membrane and nucleus, and molecular functions including nucleotide binding, metal ion binding, transferase, kinase, and oxidoreductase activity involved in biological processes such as phosphorylation, oxidation–reduction, cell redox homeostasis process, and DNA repair. Compared to the susceptible genotype which showed significant up-regulation of genes involved in processes like DNA repair, the significantly up-regulated DEGs of the resistant genotypes were involved in processes like energy metabolism and environmental adaptation, particularly host-pathogen interaction. This indicates an efficient utilization of environmental adaptation pathways, energy homeostasis, and stable DNA molecules as the strategy to cope with *Fusarium* wilt infection in chickpea. The findings of the study will be useful in targeting the genes in designing gene-based markers for association mapping with the traits of interest in chickpea under *Fusarium* wilt which could be efficiently utilized in marker-assisted breeding of chickpea, particularly against *Foc4 Fusarium* wilt.

## 1. Introduction

Chickpea (*Cicer arietinum* L) is a protein and nutrient-enriched pulse crops, cultivated in different parts of the world, with an area of about 15 million hectares (Mha) and an annual production of 16 million tons (MT; FAOSTAT, 2023). India contributes more than 75% of the world’s chickpea production (around 12 MT @ 1,192 kg ha^−1^) in an area of around 10 Mha (Indiastat, 2023). The demand for protein and other nutrient-dense foods like pulses is increasing to meet the food and nutritional security of the growing world population. However, the area and production of chickpea have only increased marginally in the past few decades. One of the reasons for the antagonistic production and productivity of chickpea is *Fusarium* wilt disease, caused by *Fusarium oxysporum* f. sp. *ciceris (Foc). Fusarium* wilt is a devastating biotic stress that causes up to 100% yield loss in chickpea (Jendoubi et al., 2017) if the proper disease management practices are not followed.

The *Fusarium* wilt disease is very difficult to manage by conventional methods like the application of fungicides, crop rotation, etc., as the nature of the pathogen is soil- and seed-borne and it can survive in the soil for up to 6 years without a host (Haware et al., 1996). However, most of the time, farmers’ preference is higher yield over disease resistance. Several high-yielding varieties that are preferred by farmers are susceptible to *Fusarium* wilt. Hence, the cultivation of wilt-resistant varieties of chickpea is the best way to overcome yield and production losses. Cultivation of one resistant variety over a long time in the same field may also lead to a breakdown of the resistance mechanism by pathogens, as many of these mechanisms are controlled by one major gene or oligogenes (Adugna, 2004). The different races of this pathogen cause wilt disease and other related symptoms in chickpea across chickpea-growing countries. The wilting symptom in chickpea is caused by specific races of this pathogen, namely 1A, 2, 3, 4, 5, and 6 (Jendoubi et al., 2017).

Therefore, understanding the molecular mechanisms behind the resistance to *Fusarium* wilt is a positive step toward the development of chickpea genotypes with better yield and quality, along with resistance to diseases. To understand the underlying molecular mechanisms in chickpea plants for resistance against *Fusarium* wilt, various approaches, including “omics” tools, are being utilized, such as cDNA-RAPD and cDNA-AFLP techniques (Gupta et al., 2009), small RNA sequencing to identify microRNAs (Kohli et al., 2014), genome-wide transcriptome profiling (Sharma et al., 2016), and a long SAGE transcriptome approach to understanding host-pathogen interaction (Upasani et al., 2017). Similarly, studies on root transcriptome, marker-trait association, marker-based DNA profiling, and race-specific marker development against several races of *Foc* were conducted (Yadava et al., 2023) and these reported several underlying genes and pathways involved in host-pathogen interaction and resistance mechanisms. However, most of these studies were conducted with races 1, 2, 3, and 5 of *Foc* and with different genotypic backgrounds of the chickpea.

Considering the above burning issues, the present study was conducted with two contrasting genotypes of chickpea (one highly resistant and one highly susceptible) against *Foc4,* aiming to reveal the important molecular pathways that are associated with *Fusarium* wilt resistance and the genes involved in it. The identified genes could be targeted to design candidate gene-based markers to be utilized in marker-trait association (MTA) studies. The MTA studies will enable us to understand how these genes are directly or indirectly imparting resistance against *Fusarium* wilt via different traits; that information will ultimately help to develop chickpea genotypes by using marker-assisted breeding, particularly against *Foc4*.

## 2. Materials and methods

### 2.1. Experimental details

Two chickpea genotypes, namely KWR 108 and GL 13001, were selected for the study as these two varieties were reported to be highly contrasting in response to *Fusarium* wilt (Kumar et al., 2019), particularly to *Foc4*. The experiment was conducted in the *Fusarium* wilt sick plot of TCA, Dholi farm (a designated plot for chickpea *Fusarium* wilt pathological trials from the All India Co-ordinated Research Projects on Chickpea located at TCA-Dholi under the Department of Agricultural Biotechnology and Molecular Biology, CBS&H, Dr. Rajendra Prasad Central Agricultural University, Pusa (Samastipur) 848,125, Bihar, India). *F. oxysporum* isolates were collected from the field of TCA, Dholi and identified as *Foc4* (Sharma et al., 2014). Among these chickpea genotypes, KWR 108 was reported as highly resistant and GL 13001 was reported as a highly susceptible genotype against *Fusarium* wilt (Kumar et al., 2019). The seeds of KWR 108 and GL 13001 genotypes were procured from the ICAR-Indian Institute of Pulse Research, Kanpur, Uttar Pradesh, India. Seeds of both genotypes were sown in a grow bag filled with a mixture of autoclaved soil and compost at a ratio of 6:4. For each genotype, two seeds were sown in each grow bag. After germination, only one plant bag^−1^ was maintained for 25 days after sowing. Then plants of both genotypes were divided into two groups, namely control and treatment, which had three biological replicates. All the field experiments were carried out under the net house condition during rabi season 2021–2022, and the molecular works were carried out at the Functional Genomics Laboratory of the Department of Agricultural Biotechnology and Molecular Biology (25.98°N latitude and 85.67°E longitude and 52 m altitude from mean sea level), College of Basic Sciences and Humanities, Dr. Rajendra Prasad Central Agricultural University, Pusa 848,125, Bihar, India during the year 2021–2022.

### 2.2. Treatment of plants with *Fusarium oxysporum* f. sp. *ciceris* culture

**Figure 1:**
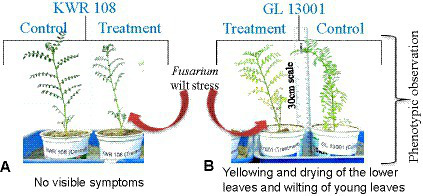
Phenotypic observations of **(A)** No visible symptoms in genotype KWR 108 and **(B)** Yellowing and drying of lower leaves and wilting of young leaves in genotype GL 13001 due to the infection of *F. oxysporum* as compared to control.

For infecting the plants, broth culture was prepared from the pure culture plates of *Foc4* (pure culture was derived from the wilt-infected chickpea plant grown at the wilt sick plot of TCA, Dholi). Small-size inoculums (0.5 cm^2^) from the pure culture plate were inoculated in broth culture media (Potato Dextrose Broth) with the help of a forcep. The broth culture was incubated at 26°C and 150 rpm in a rotary agitator for 7 days. For inducing wilt infection in the treatment group of plants, the broth culture was thoroughly mixed using a pipette, and 2 mL of the solution was added to each plant at the root zone with the help of a pipette. To further ensure the infection of plants in treatment groups, soil collected from the *Fusarium* wilt sick plot of TCA Dholi was filled on the top of the grow bags (treatment), followed by light irrigation. Symptoms appeared in the susceptible genotypes within 8–10 days of the treatment (Figures 1A,B). The plant leaf samples were collected after 10 days of treatment for transcriptome sequencing.

### 2.3. RNA isolation and preparation of library for transcriptome sequencing

The leaf samples were collected from control and treated plants of both the genotypes in three biological replicates and immediately dipped in the 2 mL micro-centrifuge tubes filled with RNA stabilizer solution (G-Biosciences) and outsourced for RNA isolation and transcriptome sequencing at Unigenome, Ahmedabad, Gujarat, India-380015. The leaf samples from three biological replicates were bulked together for RNA isolation. RNA was isolated by using the Alexgen Total RNA kit. RNA quality and quantity were analyzed using 1% agarose gel and Qubit^®^ 4.0 fluorometer, respectively. The NEBNext^®^ Ultra^™^ RNA Library Prep Kit for Illumina NEB #E 7770 was used for paired-end sequencing library preparation. The mRNA enrichment was performed as per the user manual and mRNA was first fragmented, followed by cDNA synthesis and repairing of ends and adenylation of 3′ end. Subsequently, the adapter was ligated at the fragmented ends and PCR amplification was performed for selective enrichment of adapter ligated DNA fragments. The adapter-based selectively enriched amplified fragments/libraries were analyzed on TapeStation 4,150 (Agilent using RNA ScreenTape^®^ as per manufacturer’s instructions). The average size of libraries was observed to be 331 bp, 363 bp for GL 13001 (control and treatment) and 353 bp and 344 bp for KWR 108 (Control and Treatment), respectively. The final library was pooled with other samples, denatured, and loaded onto the flow cell. On the flow cell, cluster generation and sequencing were performed using the Illumina Novaseq 6,000 platform to generate 2 × 150 bp paired-end (PE) reads. Data were filtered to remove the adapter and low-quality reads. The raw transcriptome sequencing data obtained from both genotypes under control and *F. oxysporum* f. sp. *ciceris*-treated plants with two technical replicates were submitted to the SRA (Sequence Read Archive) database of NCBI (accessed on 20 October 2023).^1^

### 2.4. Reference genome-based transcript mapping, assembly, and further bioinformatics works

From the NCBI database (accessed on 20 October 2023),^2^ the reference genome of *Cicer arietinum* (GCA_000331145.1_ASM33114v1) and reference genome annotation GTF file were downloaded. Subsequently, by using STAR (v2.7.10a) aligner, reference genome-based assembly of transcripts was performed first by separately mapping high-quality (HQ) clean reads of control and treatment samples of GL 13001 and KWR 108 on the reference genome, respectively. The STAR Genome-Generate mode option was used to carry out indexing of the reference genome. Then the input reads, in FASTQ format, along with the indexed reference genome generated in the previous step, were given to the STAR aligner. To identify the positions of the origin of the reads, the STAR aligner created the alignment in BAM format for each sample through reference genome-guided mapping of the HQ reads followed by assembly of transcripts using StringTie (v2.2.1). After the assembly of transcripts by StringTie, the quantification of assembled transcripts was performed. The quantification of the assembled transcripts was done in order of most highly abundant transcripts to less abundant by using the network flow algorithm. The annotation of assembled transcripts was performed using the GFF annotation file of reference genome having “known” gene exon structures followed by quantification of the expression of known genes. Simultaneously, to merge the gene structure found in all four samples, the assembled transcripts of all four samples were passed to StringTie’s merge function to avoid assembly of only a partial version of any of the transcripts in the initial StringTie run.

### 2.5. Identification of novel transcripts and novel isoforms of transcripts

The reference GTF file and the string-tie merged GTF file were taken and the GFF compare utility was run to check or identify if there were any novel transcripts or novel isoforms of transcripts were present in the merge sample. Novel transcripts were then identified using a method described by Gleeson et al. (2022). The transcripts that did not have any available information in the annotation file of the reference genome were considered novel transcripts.

### 2.6. Differential gene expression analysis

Using the SringTie merge function, a consensus set of transcripts was derived by merging the structures of all the genes found in all four samples and simultaneously estimating the abundances of the merged transcripts in the four samples. The read count information that was obtained from the files generated by StringTie was extracted by using prepDE.py, a Python program (Gill and Dhillon, 2022). The edgeR package (Robinson et al., 2010) was then used to calculate differential gene expression (DEGs) by taking read count results of a Python program prepDE.py as input.

### 2.7. Gene ontology and KEGG enrichment pathway analysis

Gene ontology (GO) analysis was done to identify the distribution of significant differentially expressed genes (DEGs) into three major domains, i.e., cellular component, molecular function, and biological process. GO term was assigned to each significant differentially expressed transcript using Blas2GOcli 1.4. KAAS (KEGG automatic annotation server; Moriya et al., 2007) was used for ortholog identification and mapping of significant differentially expressed transcripts to the biological pathways. The criteria used to identify significantly down and up-regulated transcripts were log2FC > 0 and *q* value<0.05 (means significantly up-regulated), and log2FC < 0 and *q* value <0.05 (means significantly down-regulated). Using BLASTX with a threshold bit-score value of 60 (default), significant differentially expressed transcripts were compared against the KEGG database. A Heatmap of DEGs was generated for 25 highly significant down and up-regulated genes (a total top 50 transcripts) based on *q* value (i.e., least *q* value).

### 2.8. Identification of genes encoding differentially expressed transcripts

The reference of the genome-based transcript identification revealed that many of the transcripts showed differential expression under *Fusarium* wilt infections, which are the transcript fragments encoding the same proteins. Further analysis was conducted using the NCBI gene database. However, the protein IDs of the transcripts revealed that several proteins were the products/isoforms of the same genes. Using the sorting option available in MS Excel, the transcripts encoded by the same genes were sorted together to finally get the exact numbers of genes showing differential expression under *Fusarium* wilt stress in chickpea.

### 2.9. Primer design and real-time PCR analysis

From the RNA sequencing data, 15 genes that showed significant differential expression in the resistant genotype, i.e., KWR 108, were selected for real-time PCR verification of the RNA sequencing results. Primer Blast Program, available at NCBI database (accessed on 20 October 2023),^3^ was used for designing primers with CDS of 15 selected genes and CDS of Glucose-6-Phosphate Dehydrogenase (G6PD) gene as internal control (Reddy et al., 2016). RNA was extracted by using RNA isolation kit (Qiagen) from control and treatment plants of KWR 108 and cDNA was synthesized from RNA using the cDNA synthesis kit following the manufacturer’s protocol (Himedia, HiGenoMB). The gene expression analysis was done by using the cDNA template and 2× AB HS SYBR Green qPCR Mix (Thermo Scientific) in a qRT-PCR (Stratagene Mx3000P, Agilent). The details of real-time PCR primers specific to the ERF transcription factor family used in the experiment are listed in Table 1.

**Table 1:** List of qRT PCR primers specific to selected genes that showed significant up-regulation in KWR 108 in shoot transcriptome data for validation of RNA sequencing-based shoot transcriptome data.

Sl. no.	ERF gene Id	Forward primer sequence	Reverse primer sequence	Expt. A. size (bp)
1.	LOC101488294	ACACTCGTCAAGTTGCTGCT	TGAGGGTCCTTGAGAAGAAGG	98
2.	LOC101489178	CCAACGCCAACAACCTCAAC	GCCTTCTTCTAACCCCTCGG	90
3.	LOC101490960	GTCCTTGGGGGAAATACGCA	GCAGCTTCTTCAGCTGTTTGG	94
4.	LOC101494217	TCCGAAACCGGAGGTGATTG	TTTCCCCACGGTCTTTGTCT	90
5.	LOC101495836	AGTTCAGAGGCGTAAGGCAG	CCGCCGTGTTAAAAGTTCCG	102
6.	LOC101496212	GGAAATTCGCGGCGGAGATA	AAGCAAGTGCTGCTGCTTCA	93
7.	LOC101501427	ATACTGCGCTTAGCGTTGGT	CGCTCATAATCCACGACGGA	106
8.	LOC101502122	CGCTGAGATTCGTCTCCCTC	CTTAAAAGCTTCGCGGTCGT	100
9.	LOC101502737	CAGCAGAGATTCGTGACCCA	ACATCATAAGCACGTGCAGC	91
10.	LOC101504196	TCTGTCTCCGGTGGAGTTCA	ACAACGGTGGAGGTTCGTTT	106
11.	LOC101505842	GAAGATTGATACCGTTTCTGGAG	GCTGTCTGTTGTACTTCTTCCAC	110
12.	LOC101508871	CGCTTCCTCTGTTTCCTGGT	GAAGGTGGTAAACCCGACCC	100
13.	LOC101512924	AGAAGGCCATGGGGGAAATG	TAGCAGCTTCCTCAGCAGTG	100
14.	LOC101515629	GGTCAAGGAAGAACCTCAACCT	GCCCATTTTCCCCAAGGTCT	93
15.	LOC105852724	CTGCTTCCTCCTCCTCCTCT	CGGGTCGGGTGACTCATAAC	107
16.	G6PD	GCAATTTGCAACACCTTAGTGG	GTGGTTGAACAACTTCAGCGT	83

Expt. A. size (bp) is the expected amplicon size (base pair).

### 2.10. Data analysis and graphing

Sample comparison was made for GL 13001 (control vs. treatment) and KWR 108 (control vs. treatment), respectively, for differential expression analysis. The analyses were performed in the edge R package to identify differentially expressed genes. Reads per million mapped reads or Counts per million mapped reads for both control and treatment samples, LogCPM (Log10 of CPM value), Log2Fold Change [the logarithm (base 2) of the fold change], pval (value of *p* for the statistical significance of this change), FDR [FDR adjusted value of *p* (q-value)], and the criteria used to identify significantly down and up-regulated transcripts were log2FC > 0 (means up-regulated), log2FC > 0 and *q* value<0.05 (means significantly up-regulated), log2FC < 0 (means down-regulated), log2FC < 0 and *q* value <0.05 (means significantly down-regulated). In real-time PCR analysis, the mean and standard deviation were calculated based on three pieces of biological replicated data and, using the Livak method (Schmittgen and Livak, 2008), the relative gene expression was calculated with G6PD as the internal reference gene. The rest of the data statistics and graphing were performed using MS Excel. The reliability of the RNAseq data was confirmed by comparing the expression data of selected genes using a linear regression model in Excel.

## 3. Results

### 3.1. Transcriptome data generation, transcript mapping, and assembly

In the present investigation, the total reads obtained from four samples, i.e., GL 13001 (control and treatment) and KWR 108 (control and treatment), ranged from 33.19 million reads in GL 13001-Control to 42.43 million reads in KWR 108-Treatment (Table 2).

**Table 2:** The transcriptome data statistics (A) for clean data after removal of the adapter and low-quality bases.

Sample name	Total reads (R1 + R2)	Total bases (R1 + R2)	Total data (~Gb)
GL 13001 (Control)	33,192,474	4,94,41,97,791	4.94
GL 13001 (Treatment)	37,251,762	5,55,45,96,976	5.55
KWR 108 (Control)	35,184,976	5,24,08,35,996	5.24
KWR 108 (Treatment)	42,432,918	6,30,99,29,526	6.31

R1 and R2, two technical replicates; Gb, Gigabase.

The QC passed reads were then subjected to reference-based transcript assembly. The genome size for *Cicer arietinum* is 531 Mb. There are a total of 30,236 genes and 220,289 CDS as per the GFF file of the NCBI chickpea reference genome. The transcript assembly was performed by mapping clean reads of control and treatment samples of GL 13001 and KWR 108 on the reference genome using STAR (v 2.7.10a). The mapping statistics of HQ reads against the reference genome are presented in Table 3.

**Table 3:** Highlights of mapping statistics high-quality reads derived from shoot transcriptome mapped to chickpea reference genome using STAR aligner.

Sample name	Total reads	Mapped reads (no.)	Mapped read (%)	Uniquely mapped reads (no.)	Uniquely mapped reads (no.)
GL 13001 (Control)	33,192,474	29,327,660	88.36	27,482,244	82.80
GL 13001 (Treatment)	37,251,762	35,757,750	95.99	33,881,974	90.95
KWR 108 (Control)	35,184,976	33,512,528	95.25	32,497,028	92.36
KWR 108 (Treatment)	42,432,918	40,655,068	95.81	23,111,318	54.47

The number of assembled transcripts varied from 36,081 to 39,186 with an average transcript size of 2003.40 bp and 1992.60 bp in control and treated GL 1300, respectively. Similarly, the number of assembled transcripts ranged from 37,496 and 37,304 with an average transcript size of 2005.90 bp and 1984.60 bp in control and treated KWR 108 leaves samples, respectively. Simultaneously, StringTie’s merge resulted in several transcripts assembled as 51,353 with an average transcript size of 1898 bp in merge data (Table 4).

**Table 4:** The statistics of merged transcripts (generated by the combination of all four samples as well as reference genome) and individual transcript assembly using StringTie assembly and merge function.

Sample name	Assembled transcripts (no.)	Total assembled (bp)	Mean transcript size (bp)	Max transcript size (bp)
Merged	51,353	9,74,67,567	1,898	33,235
GL 13001 (Control)	36,081	7,22,83,697	2,003.40	33,235
GL 13001 (Treatment)	39,186	7,80,82,916	1,992.60	33,235
KWR 108 (Control)	37,496	7,52,13,757	2,005.90	33,235
KWR 108 (Treatment)	37,304	7,40,32,603	1,984.60	33,235

StringTie’s merge function was used because individually in some of the samples, transcripts might only be partially covered by reads, leading to the assembly of only a partial version of those transcripts in the initial StringTie run. Thus, the set of transcripts that are consistent in all four samples was created to be compared in the subsequent steps using the merge step.

### 3.2. Novel transcripts and novel isoform transcripts identified

A total of 4,111 novel transcript isoforms corresponding to or encoded by 2,918 genes were identified as potential novel isoforms. Interestingly, 167 novel transcripts in KWR 108 and 170 novel transcripts in GL 13001, which were not available in the annotation file of the reference genome, were identified. The detailed list of novel transcripts and novel isoform transcripts identified are enlisted in Supplementary raw data file S1.

### 3.3. Identification of differentially expressed transcripts

Large numbers of transcripts were shown to be differentially expressed as revealed by differential expression analysis using edgeR software. Simultaneously, 7,504 and 6,698 transcripts were found to be significantly differentially expressed in GL 13001 and KWR 108, respectively (Table 5).

**Table 5:** The statistics of transcripts showing differential expression.

Transcripts combinations	Total transcripts	Down-regulated	Up-regulated	Significant-down-regulated	Significant-up-regulated	Total significant transcripts
GL 13001 (Treatment vs. Control)	32,430	15,428	17,002	2,835	4,669	7,504
KWR 108 (Treatment vs. Control)	31,038	15,789	15,249	3,139	3,559	6,698

The statistics of calculating up and down-regulated genes were log2FC > 0 (up-regulated), log2FC < 0 (Down-regulated), log2FC > 0 and *q* value < 005 (significantly up-regulated) and log2FC < 0 and *q* value < 0.05 (significantly down-regulated).

**Figure 2:**
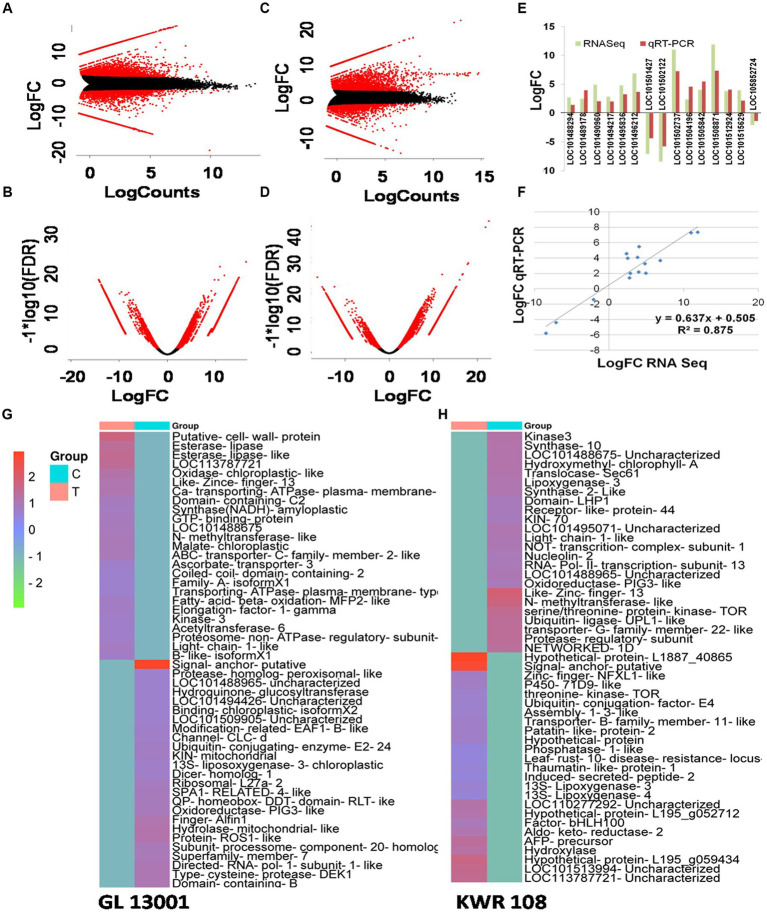
MA plot and volcano plot showing differentially expressed transcripts in treatment vs. control combination in GL 13001 **(A,B)** and KWR 108 **(C,D)**. Red corresponds to transcripts with adjusted *p* value/*q* value <0.05. Validation of RNA-Seq data by qRT-PCR by using primers specific to 15 genes which in RNA-Seq result showed significant differential expression in KWR 108 **(E,F)**. The heatmap of 25 highly significant down and up-regulated transcripts based on *q* value (i.e., least *q* value) in GL 13001 **(G)** and KWR 108 **(H)**. The color coding ranges from red to green, where shades of red represent high transcript expression and shades of green represent low transcript expression. T means treatment sample and C means control sample.

By transforming the data onto A (mean average) and M (log ratio) scales, the differences between measurements taken in control and treatment samples were visualized in MA plot. Simultaneously, a volcano plot that arranges differentially expressed transcripts along dimensions of statistical as well as biological significance was obtained from the edgeR software (Figures 2A–D). Similarly, the heatmap of 25 highly significant down and up-regulated genes (total 50 transcripts) based on q-value (i.e., least *q* value) and transcripts with proper annotations generated for both the genotypes are represented in Figures 2G,H.

### 3.4. Validation of RNAseq-based transcriptome data using a real-time PCR

The reliability of sequencing data was confirmed by real-time-based expression analysis of 15 selected genes that showed significant up-regulation in the resistant genotype under *Fusarium* wilt infection as revealed by RNAseq data. Similar to RNA sequencing results, the real-time PCR data of 15 selected genes showed significant differential expression of the genotype KWR 108 (Figure 2E). The linear regression analysis of RNAseq and real-time PCR-based expression data also showed significant similarity (*R^2^* = 0.871), which confirmed that the RNAseq data were reliable for further analysis (Figure 2F).

### 3.5. Reference genome-based transcript identification

Identification of genes encoding differentially expressed transcripts revealed that, in GL 13001, 427 genes were significantly down-regulated and 794 were significantly up-regulated (Supplementary raw data file S2). Similarly, in KWR 108, 495 genes were significantly down-regulated and 608 genes were significantly up-regulated (Supplementary raw data file S2). When the genes that showed significant differential expression were compared between two genotypes, i.e., GL 13001 and KWR 108, it was revealed that 81 genes were down-regulated and 211 genes were up-regulated in both the genotypes. Ninety-one and 172 genes showed significant down-regulation and up-regulation only in GL 13001 and 93 and 97 genes showed significant down-regulation and up-regulation only in KWR 108, respectively. Similarly, 111 genes were significantly up-regulated in GL 13001 but significantly down-regulated in KWR 108 and 50 genes were significantly down-regulated in GL 13001 but significantly up-regulated in KWR 108.

### 3.6. Gene ontology enrichment analysis

**Figure 3:**
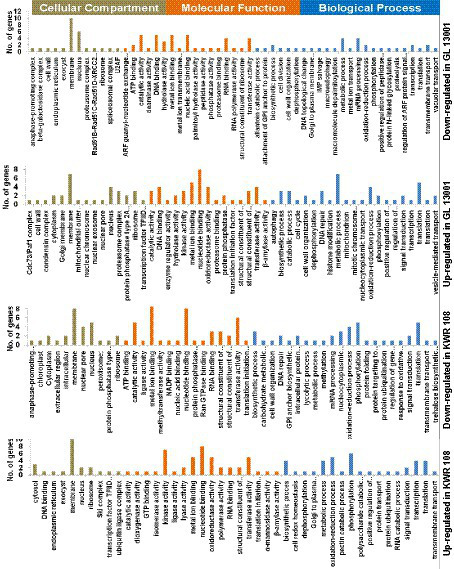
Gene ontology (GO) enrichment graphs of the top 50 up and 50 down-regulated genes in KWR 108 and GL 13001. Moss green color bar represents Cellular Compartments, Orange color bar represents Molecular functions, and Blue color bar represents biological processes in which the top 50 DEGs are involved.

The GO enrichment analysis of significant differentially expressed genes (DEGs) summarized the genes into three main GO categories, namely cellular component, molecular function, and biological process (Supplementary raw data file S2). The GO enrichment results of the top 50 significantly up-regulated and down-regulated genes in two genotypes are represented as pie charts (Figure 3).

The GO enrichment results of the top 50 significantly up and down-regulated genes revealed that the proteins encoded by most of these genes are the components of the membrane and nucleus. The products of most of the top 50 genes down-regulated in GL 13001 have nucleotide binding, catalytic, metal ion binding, transferase, and hydrolase activity involved in biological processes like metabolic processes, mRNA processing, DNA repair, oxidation–reduction process, and macroautophagy. Apart from membrane and nucleus, many of the proteins encoded by the top 50 genes up-regulated in GL 13001 are the components of proteasome and ribosome having nucleotide binding, catalytic, metal ion binding, oxidoreductase, and transferase activity involved in biological processes like metabolic processes, translation, DNA repair, and oxidation–reduction process.

Similar to the GO terms of the top 50 genes significantly up and down-regulated in GL 13001, the GO terms of the top 50 genes down-regulated in KWR 108 showed the highest cellular component as membrane and nucleus and molecular functions as nucleotide binding, metal ion binding, transferase, catalytic, and nuclear pore activities. However, the GO terms of the top 50 genes up-regulated in KWR 108 revealed that, apart from membrane and nucleus, the products of the genes are the components of ribosome and cytosol having molecular functions such as kinase activity, oxidoreductase activity, nucleotide binding, transferase, and metal ion binding activities involved in biological processes such as phosphorylation, oxidation–reduction process, metabolic and biosynthetic process, and cell redox homeostasis (Figure 3).

### 3.7. Pathways involving differentially expressed genes

**Figure 4:**
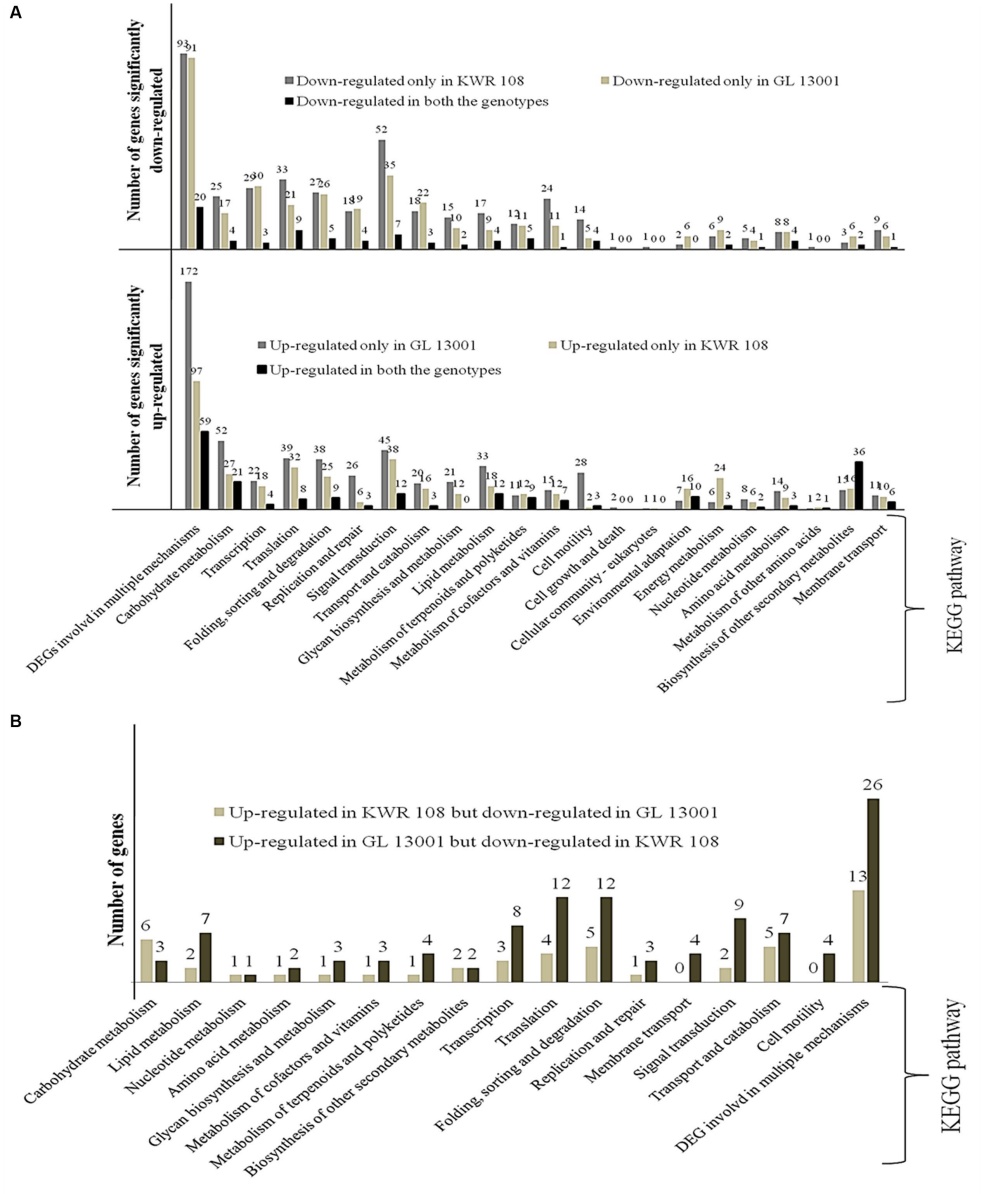
KEGG enrichment/Pathway analysis results **(A)** genes significantly up-regulated and down-regulated in two genotypes of chickpea, i.e., KWR 108 and GL 13001 and **(B)** genes significantly up-regulated in KWR 108 but down-regulated in GL 13001 and vice versa. ‘Y’ axis represents the number of significant DEGs.

KEGG enrichment analysis of the significant DEGs identified in both the genotypes of chickpea was performed to estimate the numbers of significant DEGs involved in different KEGG pathway levels. It revealed that, in the susceptible genotype, i.e., GL 13001, the number of significantly up-regulated genes involved in different pathways such as DNA replication and DNA repair, cell motility, lipid metabolism, carbohydrate metabolism, and DNA degradation is significantly higher than in KWR 108. Whereas the number of DEGs specifically involved in energy metabolism and environmental adaptation pathways are significantly higher in KWR 108 than in GL 13001 (Figure 4A). The same genes that were up-regulated in GL 13001 but down-regulated in KWR 108 were observed to be higher in most of the pathways; more specifically, the genes involved in cell motility and membrane transport were significantly up-regulated only in GL 13001 (Figure 4B). However, the same genes involved in carbohydrate metabolism were up-regulated in resistant genotype KWR 108 but down-regulated in susceptible genotype GL 13001 (Figure 4B).

## 4. Discussion

In the present study, transcriptome dissection was done to understand the probable molecular mechanisms imparting the ability to tolerate *Fusarium* wilt in the chickpea genotype, particularly in the genome background of KWR 108, a highly resistant variety screened at TCA-Dholi, Bihar, India (Kumar et al., 2019). The comparative analysis of the transcriptome data generated from susceptible genotypes GL 13001 and resistant variety KWR 108 was performed to identify the pathways and genes involved that are showing differential expression under *Fusarium* wilt stress. Compared to the susceptible genotypes, higher numbers of genes belonging to the pathways involved in environmental adaptation and energy metabolism showed highly significant up-regulation in resistant genotype KWR 108 and, along with genes involved in several other processes, more genes belonging to DNA repair mechanisms showed significant up-regulation in susceptible genotype GL13001 (Figure 4A).

### 4.1. Significant up-regulation of genes involved in DNA repair mechanism in susceptible genotype

In our present study, a comparison between the shoot transcriptome of KWR 108 and GL 13001 showed a significantly higher number of genes involved in DNA repair mechanisms were up-regulated in GL 13001, a susceptible genotype (Figure 4A). Although, under normal circumstances, DNA damage can be thought of as a frequent occurrence, it is likely to occur even more in response to several stress conditions (Nisa et al., 2019) either directly or indirectly due to increased level of reactive oxygen species (ROS; Szurman-Zubrzycka et al., 2023). The resistant or tolerant plants protect the DNA from damage by activating enzymatic and non-enzymatic antioxidant machinery (Moradbeygi et al., 2020). In the present study, the significantly up-regulated genes related to DNA repair mechanism in susceptible genotypes are crossover junction endonuclease EME1, DNA excision repair protein ERCC-1 & 4, DNA mismatch repair protein MLH3, DNA repair and recombination protein RAD54 and RAD54-like protein, DNA repair protein REV1, DNA-3-methyladenine glycosylase I, flap endonuclease-1, telomere length regulation protein, UV excision repair protein RAD23, and WD repeat-containing protein 48. This suggests that DNA replication and other similar processes of DNA synthesis are more stable in resistant genotypes as compared to susceptible genotypes under *Fusarium* wilt stress condition.

### 4.2. Significant up-regulation of environmental adaptations genes in the resistant genotype

The genes specifically belonging to environmental adaptation pathways are significantly up-regulated in the resistant genotype. The environmental adaptation genes basically encode for the proteins or enzymes involved in plant-pathogen interaction upon infection. In the present study, the significantly up-regulated environmental adaptation genes in KWR 108 were calcium-dependent protein kinase (CDPK), pathogenesis-related genes transcriptional activator (Pti5 & Pti6), calmodulin (CaM), calmodulin-like proteins (CML), cyclic nucleotide-gated channel (CNGC), mitochondrial translation elongation factor Tu (TUFM), chitin elicitor receptor kinase 1 (CERK1), enhanced disease susceptibility 1 protein (EDS1), disease resistance protein (RPM1/ RPS3), mitogen-activated protein kinase 3 (MPK3), mitogen-activated protein kinase 4 (MPK4), mitogen-activated protein kinase kinase 4/5 (MKK4_5), pathogenesis-related protein 1 (PR1), and WRKY transcription factor 33 (WRKY33). The important roles and involvement of environmental adaptation genes like CDPK (Asano et al., 2012; Baba et al., 2019; Wu et al., 2021), Pti5 & Pti6 (Yang et al., 2020; Wang Y. et al., 2021; Sun et al., 2022), CaM, and CML (Zeng et al., 2015; Ranty et al., 2016) in several biotic and abiotic stress responses have been extensively studied and reported. The plant accumulates Ca^+^ upon experiencing stress as a secondary messenger to modulate the cellular processes (Luan, 2009). CNGC, which showed significant up-regulation under *Fusarium* wilt in the present study, is mostly localized to the plasma membrane and acts as ligand-gated cation channels (Chin et al., 2009) for the accumulation of Ca^+^ in the cell during stress. Several reports suggested the potential role of CNGC in plant disease resistance through hypersensitive response (Clough et al., 2000; Ahn, 2007; Keisa et al., 2011; Ma and Berkowitz, 2011; Guo et al., 2018; Peng et al., 2019; Dietrich et al., 2020; Chakraborty et al., 2021; Zhao et al., 2021). Mitochondrial translation elongation factor Tu (TUFM) is a mitochondrial protein encoded by the nuclear genome that potently inhibits RLR (RIG-I-like receptors) signaling and promotes autophagy during a pathogen infection, particularly viruses (Lei, 2011; Wu et al., 2022). In the study, TUFM showed significant up-regulation in *Fusarium* wilt-resistant genotype KWR 108, suggesting its potential role in imparting resistance against fungal pathogens and other stresses as reported in crops like maize (Rao et al., 2004) and soybean (Yin et al., 2009). Chitin, which is an essential unit of fungal cell walls including chickpea wilt, causes *F. oxysporum* to act as an elicitor of plant immunity (Gong et al., 2020). Chitin elicitor receptor kinase 1 (CERK1), which showed significant up-regulation in resistant genotypes in the present study, is a heterotrimeric complex (Gigli-Bisceglia and Testerink, 2021) required for the perception of chitin and subsequent activation of the chitin-responsive cellular processes as well as for triggering the innate immunity of the plants against abiotic stress (Espinoza et al., 2017). CERK1 mutant (cerk1) plants showed increased susceptibility to fungal pathogens like *Erisiphe cichoracearum, Alternaria brassicicola*, and *Blumeria graminis* (Gigli-Bisceglia and Testerink, 2021), indicating its vital role in perceiving pathogen signals and subsequent induction of defense mechanism in resistant plants. The enhanced disease susceptibility 1 (EDS1) gene, which showed significant upregulation in KWR 108 in the present study, encodes a lipase-like protein that has been reported to be essential for resistance against biotrophic pathogens (Ochsenbein et al., 2006). In plants, it is basically required by Toll/Interleukin-1 receptor (TIR) domain of NLR (nucleotide-binding leucine-rich repeat) immune receptors (Johanndrees et al., 2023) and for inducing expression of PR (pathogen-related) proteins through the salicylic acid (SA) pathway against several pathogens (Yan et al., 2016; Bhandari et al., 2019; Neubauer et al., 2020). Likewise, RPM1/RPS3 (disease resistance protein), which in the present study showed significant up-regulation in the resistant genotype, is basically a CC-NBS-LRR protein that was first identified as a protein in Arabidopsis essential for resistance mechanism against *P syringae pv. Maculicola*. It was later reported to have roles in resistance against diseases caused by other pathogens like *Puccinia striiformis f.* sp. *tritici* through hypersensitive response and Ca^+^ concentration dynamics (Grant et al., 2000; Wang et al., 2020). In the present study, mitogen-activated protein kinases (MAPK) such as MPK3, MPK4, and MKK4_5 showed significant up-regulation in resistant genotypes that are basically involved in intracellular responses (Sinha et al., 2011). In plants, MAPKs are reported to induce stress tolerance (Lin et al., 2021) including biotic stresses (Zhang and Zhang, 2022) through JA (Jasmonic acid) and SA signaling pathways (Jagodzik et al., 2018). The SA signaling in plants under stress including fungal infection induces the expression of defense proteins like PR1 (pathogenesis-related protein 1; Almeida-Silva and Venancio, 2022; Anuradha et al., 2022). Antifungal and several other antimicrobial activities of PR1 have been well-reviewed and reported (Kattupalli et al., 2021; Almeida-Silva and Venancio, 2022; Anuradha et al., 2022; Pečenková et al., 2022). Similar to all these reports, in the present study also, PR1 genes showed significant up-regulation in chickpea under *Fusarium* wilt stress, confirming its role as a defense mechanism against *Fusarium* wilt disease. Likewise, in plant defense responses, an important oxidoreductase enzyme is peroxidase, which is involved in cell wall modifications and the generation of reactive oxygen species (ROS; Chang et al., 2021). Xue et al. (2017) reported that, in common bean, the peroxidase gene PvPOX1 enhanced resistance against *Fusarium* wilt. In the present study, a large number of peroxidase genes up-regulated in KWR 108, but none in GL 13001, suggesting its role in resistance against *Fusarium* wilt in chickpea also. Similarly, WRKY33, an important member of WRKY transcription factor family in plants, showed significant up-regulation in the resistant genotype. The role of WRKY transcription factor has been studied extensively and it plays a role in responses to several stimuli irrespective of physiological, biotic, or abiotic stress (Phukan et al., 2016; Cheng et al., 2021).

### 4.3. Significant up-regulation of energy metabolism genes in the resistant genotype

The important metabolites involved in redox homeostasis of plant cells are nicotinamide adenine dinucleotide (NAD) and its other derivatives, which are mostly nucleotide coenzymes (Kapoor et al., 2015). The redox state of NAD, i.e., NAD (P)+/NAD(P)H (ratio of oxidized to reduced form) in the cell plays an important role as a signal to ameliorate the bridge between cellular metabolic state and gene expression under various conditions, including biotic and abiotic stress condition (Kapoor et al., 2015), and are vital for homeostasis of cellular energy in plant cells under stress condition for optimum growth and development. In the present study, genes related to NAD (P)H, such as NADH-dependent glutamate synthase, NAD(P)H-quinone oxidoreductase subunit 4, NADH dehydrogenase (ubiquinone) Fe-S protein, NADH:quinone reductase, and NADH–ubiquinone oxidoreductase, showed significant up-regulation in resistant genotypes, confirming its role in energy homeostasis in plants under stress conditions. Other than NADH-related genes, shoot transcriptome data of resistant genotypes in the present study also showed significant up-regulation of other genes involved in energy metabolism, such as phosphoenolpyruvate carboxylase (Wang et al., 2016; Waseem and Ahmad, 2019), alcohol dehydrogenase (Su et al., 2020), alanine transaminase (Bashar et al., 2020), ATP citrate (pro-S)-lyase (Liu F. et al., 2022), ATPase (Li J. et al., 2022; Li Y. et al., 2022), carbonic anhydrase (Rudenko et al., 2021), cytochrome c, cytochrome c oxidase (Guerra-Castellano et al., 2018; Analin et al., 2020), fructose-bisphosphate aldolase (Lv et al., 2017; Cai et al., 2022), malate dehydrogenase (Song et al., 2022), 6-phosphofructokinase 1 (Wang H. et al., 2021), photosystem II P680 reaction center D1 protein (Landi and Guidi, 2022), phosphoserine aminotransferase (Wang et al., 2022), pyruvate-orthophosphate dikinase (Yadav et al., 2020), and serine O-acetyltransferase (Mulet et al., 2004; Liu D. et al., 2022), and their molecular mechanism and role in energy homeostasis in different crops is well reviewed. It indicates that the significant up-regulation of the genes involved in energy metabolism in KWR 108 might have helped it to cope with *Fusarium* wilt stress by maintaining energy homeostasis in cells under stress conditions.

The genes particularly involved in environmental adaptation, energy metabolism, and others which showed significant up-regulation in wilt-resistant genotypes will be a target for designing gene-based markers for marker-trait association studies. The marker-trait association study, especially for the traits attributing to wilt resistance and yield under wilt infection, will be our next target to check whether these up-regulated genes have any roles in imparting resistance against *Fusarium* wilt through some desirable phenotypic traits or whether there are no roles in phenotypic traits of chickpea under wilt. If these gene-based marker/s are found to be robustly associated with *Fusarium* wilt-resistant traits, then they could be utilized in marker-assisted breeding to develop a wilt-resistant high-yielding variety of chickpea.

## 5. Conclusion

The present study was conducted to gain insight into the molecular mechanism imparting resistance against *Fusarium* wilt infection in chickpea using an RNA sequencing-based shoot transcriptome approach. The shoot transcriptome data derived from two contrasting genotypes of chickpea, namely KWR 108 (wilt resistant variety) and GL 13001 (wilt susceptible variety), revealed that the resistant genotype efficiently maintains the energy homeostasis in the cell under stress conditions by up-regulating the large sets of genes responsible for maintaining the cellular energy homeostasis. The up-regulation of significant numbers of genes involved in environmental adaptation, particularly host-pathogen interaction in resistant genotypes, suggests that the resistant genotypes efficiently deploy the defense mechanism starting from perceiving and transduction of pathogen signal to activation and implementation of the defense-related mechanism. Similarly, the susceptible genotype showed significant up-regulation of genes involved in several mechanisms, including the DNA repair mechanism, thus it can be perceived that the DNA molecules of the susceptible genotype were more unstable and damaged than the DNA molecules of the resistant genotype, which showed optimum growth under *Fusarium* wilt stress. Therefore, it can be concluded that efficient energy metabolism, activation of environmental adaptation mechanisms, and DNA stability were key to resistance against *Fusarium* wilt infection in chickpea genotypes.

## Data availability statement

The datasets presented in this study can be found in online repositories. The names of the repository/repositories and accession number(s) can be found in the article/Supplementary material.

## Author contributions

KB: Conceptualization, Data curation, Formal analysis, Funding acquisition, Investigation, Methodology, Project administration, Resources, Software, Supervision, Validation, Visualization, Writing – original draft. MA: Conceptualization, Funding acquisition, Investigation, Methodology, Resources, Supervision, Validation, Visualization, Writing – original draft. AK: Conceptualization, Investigation, Methodology, Resources, Supervision, Validation, Visualization, Writing – original draft. RS: Investigation, Methodology, Validation, Visualization, Writing – original draft. NB: Methodology, Software, Validation, Visualization, Writing – original draft. VS: Investigation, Methodology, Validation, Visualization, Writing – original draft. BP: Methodology, Software, Validation, Visualization, Writing – original draft. MT: Investigation, Methodology, Validation, Visualization, Writing – original draft. OO: Data curation, Formal analysis, Funding acquisition, Software, Writing - review & editing. VB: Data curation, Formal analysis, Funding acquisition, Software, Writing – review & editing. MB: Data curation, Formal analysis, Funding acquisition, Software, Writing – review & editing. MS: Data curation, Formal analysis, Funding acquisition, Software, Writing – review & editing. AG: Data curation, Formal analysis, Funding acquisition, Software, Writing – review & editing. AH: Data curation, Formal analysis, Funding acquisition, Software, Writing – review & editing.

## Footnotes

^1^https://submit.ncbi.nlm.nih.gov/subs/sra/SUB12177877/

^2^https://www.ncbi.nlm.nih.gov/genome/?term=Chickpea

^3^https://www.ncbi.nlm.nih.gov/tools/primer-blast/
